# Oral Immunotherapy-Induced Changes in IgE, IgG, and IgA: A Review of Antibody Isotype Shifts and Their Clinical Relevance in Food Allergy

**DOI:** 10.3390/antib15010006

**Published:** 2026-01-07

**Authors:** Giovanni Lasagni, Laura Vetrugno, Chiara Maria Maggiore, Chiara Galassetti, Giulia Di Colo, Francesco Pavan, Andrea Costantino, Lorenzo Dagna

**Affiliations:** 1 Unit of Immunology, Rheumatology, Allergy, and Rare Diseases IRCCS San Raffaele, Vita-Salute San Raffaele University, IRCCS San Raffaele, 20132 Milan, Italy; vetrugno.laura@hsr.it (L.V.); maggiore.chiaramaria@hsr.it (C.M.M.); galassetti.chiaramih@hsr.it (C.G.); dicolo.giulia@hsr.it (G.D.C.); dagna.lorenzo@unisr.it (L.D.); 2 Department of Pathophysiology and Transplantation, University of Milan, 20122 Milan, Italy; francesco.pavan@unimi.it (F.P.); 3 Gastroenterology and Endoscopy Unit, Foundation IRCCS Ca’ Granda Ospedale Maggiore Policlinico, 20122 Milan, Italy; andrea.costantino@policlinico.mi.it

**Keywords:** food allergy, immunoglobulin, immunotherapy, IgE, IgG, IgA

## Abstract

Background: Food allergy is a growing public health concern, and oral immunotherapy (OIT) has emerged as a promising approach to induce desensitization and potentially sustained unresponsiveness to allergenic foods. Changes in humoral immunity, particularly in allergen-specific immunoglobulin levels, play a central role in the immunological mechanisms underlying OIT. This review aims to summarize the current evidence on how OIT modulates allergen-specific immunoglobulin E (IgE), G (IgG) and A (IgA) responses in individuals with food allergy. Methods: We conducted a review of original research articles reporting longitudinal data on allergen-specific IgE, IgG, and/or IgA in patients undergoing OIT for common food allergens. Results: OIT was consistently associated with a transient increase in allergen-specific IgE levels during early phases, followed by a gradual decline. In contrast, Allergen-specific IgG4 levels showed a robust and sustained increase, correlating with desensitization and proposed to function as blocking antibodies. Several studies also reported an increase in allergen-specific IgA, particularly secretory IgA at mucosal sites, suggesting a potential role in enhancing mucosal tolerance and immune exclusion of allergens. Conclusions: Humoral immune responses during OIT are characterized by distinct and dynamic changes in immunoglobulin patterns. In particular, the rise in IgG4 and, in some cases, IgA suggests a role in promoting tolerance. Monitoring these biomarkers may offer insights into treatment efficacy and support individualized approaches to OIT.

## 1. Introduction

Food allergies represent a major global health issue and their significant impact on quality of life and economic burden highlights the urgent need for more effective treatment strategies. For many years, the management of food allergies has centered on strict avoidance of allergenic foods and the use of emergency treatment following accidental exposure. This traditional paradigm is now shifting, as a growing number of therapeutic approaches are being developed and moving toward clinical use [1,2,3].

Although there is currently no definitive cure for IgE-mediated food allergies, oral immunotherapy (OIT) has emerged as a promising treatment option [4]. During OIT, small amounts of allergen are administered over time until the patient can safely tolerate larger amounts of the allergen without clinical symptoms, a process known as desensitization. This prevents allergic reactions and/or reduces their severity in the case of accidental allergen exposure. The term “tolerance” has not been used in the context of studies on food allergy immunotherapy due to the difficulty of demonstrating persistent clinical protection. Instead, the term “Sustained Unresponsiveness” (SU) has been used to describe clinical non-responsiveness to the food after a period of avoidance once immunotherapy has ended. To date, OIT is the only “disease-modifying” therapeutic strategy for food allergy [5,6].

OIT is currently indicated for patients with documented severe IgE-mediated food allergy. The European Academy of Allergy and Clinical Immunology (EAACI) currently recommend OIT starting from 4–5 years of age, as tolerance may develop spontaneously before this age. OIT has also been studied for allergens beyond these and for multiple allergies, including in combination with biologic drugs with promising results [7].

Oral immunotherapy begins with a build-up phase, during which increasing amounts of the food are administered in a hospital setting under strict medical supervision. Typically, during the build-up phase, the maximum tolerated dose is taken daily at home between dose increases, which usually occur weekly or biweekly. This phase is followed by a maintenance phase, during which the food is consumed daily at the maximum tolerated dose [6,7].

**Figure 1:**
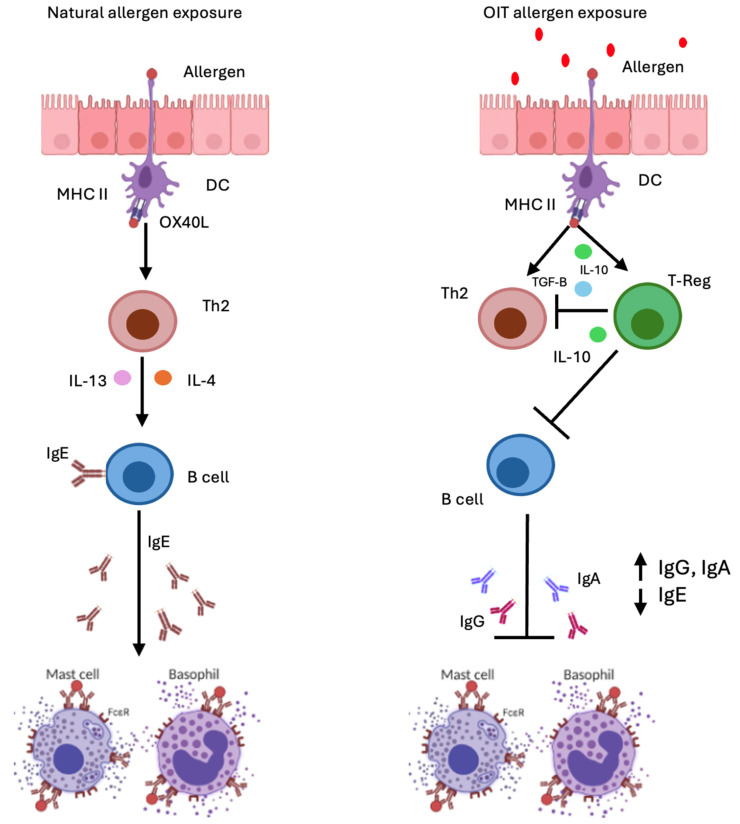
Note. In individuals with food allergy, a pre-existing Th2-skewed immune response promotes IgE class switching in B cells, leading to the production of allergen-specific IgE (sIgE). These IgE molecules bind to high-affinity Fcε receptors on mast cells and basophils, priming them for activation. Upon re-exposure to allergen doses exceeding the activation threshold, these effector cells rapidly degranulate, triggering clinical allergic symptoms. Oral immunotherapy (OIT) induces the production of IL-10 and IFN-α by dendritic cells, promoting Th2 anergy and expanding regulatory cell populations, including Tregs. These immunologic shifts reduce sIgE levels while increasing sIgG, particularly sIgG4, and sIgA. Collectively, these changes contribute to the downregulation of the allergic response and the induction of immune tolerance. DC: Dedritic cell, MHC II major histocompatibility complex, OX40L: OX40 ligand, Th2: T-cell helper 2, IL: interleukin, TGF.B: transforming growth factor beta, IgE: immunoglobulin E, IgG: immunoglobulin G, IgA immunoglobulin A.

Early phases of OIT are characterized by low-dose allergen introduction, inducing an initial increase in allergen-specific IgE (sIgE) and a concomitant expansion of type 2 effector cells and inhibition of regulatory T cell differentiation. Incremental increases in allergen exposure drive a progressive immunoregulatory response. Th2 cytokine production declines and there is increased secretion of regulatory cytokines, such as interleukin 10 (IL-10) and transforming growth factor beta (TGF-β). Allergen-specific T regulatory cells (Treg) expand and there is class switching toward IgG and IgA isotypes, which neutralize allergens by preventing allergen–IgE interactions, attenuating high-affinity IgE receptor I (FcεRI) effector cell activation, and engaging inhibitory receptors such as low-affinity receptor for Ig (FcγRIIB) that ultimately suppress basophil degranulation [8] (Figure 1).

During the maintenance phase, sustained food antigen exposure consolidates these regulatory networks. Persistent T-cell activity, together with the induction of functional T cell exhaustion that limits allergen-specific effector T cell responses, reinforces the suppression of Th2-mediated pathways, thereby maintaining clinical desensitization [9,10].

The aim of this review is to provide a comprehensive overview of the main changes in humoral immunity that occur during oral immunotherapy (OIT), with a particular focus on the dynamics of IgE, IgG, and IgA antibodies. We will explore how these immunoglobulins are modulated over the course of treatment, their roles in mediating desensitization and tolerance, and their potential as biomarkers for predicting therapeutic outcomes and guiding clinical management.

## 2. Materials and Methods

We conducted a narrative review of original research studies evaluating humoral immune responses in patients with food allergy undergoing oral immunotherapy (OIT). A comprehensive literature search was performed across multiple databases, including PubMed, Medline, and Scopus, covering articles published from September 2014 to October 2025. Search terms included combinations of “oral immunotherapy,” “food allergy,” “OIT,” “IgE,” “IgG,” “IgG4,” “IgA,” “humoral immunity,” “immunoglobulin modulation,” and “desensitization.” All identified records were screened for relevance, and reference lists of eligible articles were manually reviewed to identify additional pertinent studies.

Inclusion criteria comprised original research articles reporting longitudinal data on allergen-specific immunoglobulin responses (IgE, IgG/IgG4, and/or IgA) in individuals undergoing OIT for common food allergens. Both randomized controlled trials and observational or real-world studies were considered. Reviews, meta-analyses, case reports, and studies lacking immunoglobulin outcome data were excluded. Only studies published in English were included.

## 3. Immunoglobulin E (IgE)

IgE is the least abundant antibody isotype in human serum, with concentrations below 500 ng/mL in non-atopic subjects [11]. Despite these low circulating levels, its unique structure and domain architecture confer a uniquely elevated affinity for its primary receptor, FcεRI (equilibrium association constant ranging from 10^6^ to 10^10^ M^−1^), enabling IgE to efficiently sensitize effector cells and mediate potent immune responses at minimal concentrations [12]. Binding to effector cells extends the half-life of IgE from 2–3 days in serum to several weeks, allowing sustained sensitization of mast cells and basophils [13].

Structurally IgE is a monomeric antibody with two identical heavy (ε) chains, light chains, and a Fab region with variable domains mediating antigen recognition. Four constant domains (Cε1–Cε4) on the heavy chains lack hinge regions rendering them structurally unique from other IgG isotypes. Cε2 domains confer flexibility, allowing Fc regions to assume open or closed conformations depending on receptor or allergen engagement [14].

Cε3–Cε4 domains allow high-affinity binding of IgE to its primary receptor, FcεRI, predominantly expressed in mast cells and basophils, central to type I hypersensitivity reactions. They can also bind low-affinity IgE receptor binding (FcεRII) through the Cε3 domain regulating IgE synthesis, antigen presentation, and immune homeostasis [3,15]. Inhibitory receptors such as FcγRIIB are also expressed on mast cells. Although FcγRIIB typically engages IgG immune complexes, it can also be recruited to the mast cell surface independently of IgG, specifically under conditions of high antigen concentration where it serves to attenuate FcεRI-mediated activation [15,16].

### IgE in Food Allergy and Oral Immunotherapy

IgE-mediated food allergy in atopic individuals entails two main phases: sensitization and provocation. During sensitization, ingested allergens trigger epithelial release of TSLP, IL-25, and IL-33, activating group 2 innate lymphoid cells (ILC2s) and type 2 dendritic cells (DC). Allergen presentation to naïve CD4^+^ T cells by the type 2 dendritic cells promotes Th2 polarization, with Interleukin 4 (IL-4), 5 (IL-5), and 13 (IL-13) cytokines driving B-cell class switching, to allergen-specific IgE. Class-switch recombination to IgE requires CD40 engagement and IL-4/IL-13 driven interleukin-4 receptor (IL-4R) signaling between B and T helper cells. This cascade induces germline Cε transcription enabling recombination. High-affinity IgE is generated through somatic hypermutation and affinity maturation in germinal centers. Most high-affinity IgE are generated with sequential class switching where B cells first become IgG1+ and then undergo mutation and selection, while direct switching from IgM to IgE produces low-affinity IgE. These IgE can bind to FcεRI on mast cells and basophils, priming them for activation [17,18].

During the provocation phase, re-exposure to an ingested allergen can trigger an immediate hypersensitivity reaction through rapid crosslinking of IgE bound to FcεRI on mast cells and basophils even at minimal antigen concentrations. This high sensitivity arises in part from the affinity of IgE for its cognate allergen, describing the strength of a single antigen–antibody interaction, and more importantly from avidity, the cumulative strength of multivalent binding that predominates in vivo where allergens typically present as multivalent structures on two-dimensional surfaces. In addition, the lateral diffusion of FcεRI-bound IgE across the effector-cell membrane facilitates the formation of high-avidity IgE–allergen complexes, enabling even low-affinity IgE to achieve efficient crosslinking and robust effector-cell activation. Together, these biophysical properties explain why type I hypersensitivity reactions occur rapidly and in response to extremely small amounts of allergen [19].

While some IgE-mediated food allergies resolve with age, others (i.e., shellfish, nuts, and fish) persist, raising questions about the mechanisms underlying the maintenance of allergen-specific IgE even in the absence of significant allergen exposure. This persistence implies an immunological memory, potentially mediated by inducible class switching upon re-exposure and/or long-lived IgE-secreting plasma cells [20,21].

Oral immunotherapy is a disease-modifying approach reprogramming immunologic circuitry underlying IgE-mediated food allergy, modulating both allergen-specific IgE levels and their effector pathways. Studies show that specific IgE levels typically rise during the first months of OIT, but this early increase does not correspond to worsening allergic symptoms. With continued treatment, sIgE levels gradually decline over time, a pattern observed also in other forms of allergen immunotherapy such as sublingual or epicutaneous immunotherapy [22,23].

During OIT, IgE directed against whole allergen extracts and major components (such as seed storage proteins Ara h 1 and Ara h 2 in peanut allergy) typically increases initially and then declines over time. In contrast, IgE to minor components generally remains stable or shows only a slight decrease. This pattern suggests that IgE responses to major allergens play the most significant role in determining therapeutic outcomes. In addition, patients with a successful OIT (i.e., obtaining SU) had lower sIgE levels at baseline [24].

Total IgE has shown inconsistent patterns across clinical trials. Most studies on cow’s milk (CM), peanut, and egg immunotherapy report no significant changes in total IgE and no difference between responders and non-responders. A recent CM OIT study confirmed that total IgE at baseline or after six months does not predict treatment outcome [25,26].

Ratios of allergen-specific IgE to total IgE have been shown to predict food-challenge outcomes more accurately than specific IgE levels alone, and for this reason the sIgE/total IgE ratio has been explored as a potential biomarker during OIT. Vickery et al. showed that individuals who reintroduced peanut into their diet displayed smaller skin prick test wheals and consistently lower IgE levels to peanut, Ara h 1, and Ara h 2 at both baseline and the time of challenge [27]. They also had markedly lower peanut-specific IgE/total IgE ratios compared with those who did not pass the challenge. Together, these observations support the idea that a lower baseline load of allergen-specific IgE—reflected both in reduced skin test reactivity and in lower sIgE/IgE ratios—correlates with a higher likelihood of achieving successful peanut tolerance following OIT [27].

Furthermore, epitope-level analyses add further predictive value: testing for IgE sensitization is fundamental in the evaluation of allergic disease, yet specific IgE levels alone, while useful for estimating the probability of reactivity, do not reliably predict the severity of allergic responses. Increasing evidence shows that the qualitative features of the IgE response—particularly the specific epitopes to which IgE binds—are more clinically informative. Epitope mapping, typically performed using peptide microarrays, enhances diagnostic accuracy in food allergy, especially in patients with low sIgE levels. Across multiple studies, models incorporating IgE epitope recognition consistently outperform traditional sIgE measurements, underscoring the potential of epitope profiling to predict clinical reactivity thresholds and long-term OIT outcomes: for instance, specific Ara h 2 epitope-binding patterns strongly correlate with the likelihood of achieving sustained unresponsiveness (SU) after peanut OIT, and in milk OIT, successful desensitization is marked by reduced epitope-specific IgE and increased epitope-specific IgG4 [28,29].

This was reinforced in the POISED trial, where baseline protein- and epitope-specific IgE “fingerprints” clearly distinguished individuals who achieved SU from those with only transient desensitization, accurately forecasting long-term responses and highlighting the promise of epitope-level biomarkers for pre-treatment patient stratification [10].

Complementing these findings, a recent study demonstrated that pre-challenge salivary peanut-specific IgE correlated with reaction thresholds and severity during peanut challenge: elevated salivary IgE was associated with lower reaction thresholds and increased respiratory manifestations, whereas higher salivary IgG4 relative to IgE correlated with milder reactions. In the PPOIT-003 randomized trial, which included 162 patients ranging from 1–10 years of age who completed 18 months of peanut OIT, they found that higher baseline peanut serum IgE (sIgE) was consistently associated with a lower likelihood of remission independent of age. This again underlines that sIgE can help risk-stratify patients and inform clinical decisions about starting OIT [30]. Together, these studies suggest that both systemic and noninvasive mucosal IgE measurements could inform individualized risk assessment and guide patient selection for OIT [31].

Furthermore, to block IgE activity during OIT, the use of omalizumab has been proposed to achieve faster desensitization and reduce adverse effects. Omalizumab, an anti-IgE monoclonal antibody approved by the Food and Drug Administration (FDA) in 2024 for IgE-mediated food allergies, binds circulating IgE and lowers the expression of high-affinity IgE receptors on effector cells, thereby facilitating desensitization [32,33].

Clinical studies consistently show that omalizumab enhances the safety and feasibility of OIT. In a Phase 1 open-label trial involving patients with multiple food allergies, an 8-week omalizumab pretreatment enabled rapid multi-allergen OIT: most subjects tolerated the full dose on the first escalation day, severe reactions were rare, and all participants achieved maintenance for each allergen. Similar benefits were observed in a study combining omalizumab with milk OIT, where dose-related adverse reactions were significantly reduced during escalation, although improvements in desensitization and long-term tolerance did not reach statistical significance. Additional studies report comparable trends, reinforcing that omalizumab can facilitate faster, safer dose escalation during OIT, even in polysensitized individuals [34,35]. The main findings are summarized in Table 1.

## 4. Immunoglobulin IgG

Immunoglobulin G (IgG) is the major class of the five classes of immunoglobulins in human beings (IgM, IgD, IgA and IgE). IgG can be further divided in four subclasses, named, in order of decreasing abundance, Immunoglobulin G1 (IgG1), G2 (IgG2), G3 (IgG3), and G4 (IgG4) [36].

The “Y”-shaped structure of IgG is made up of two identical heavy chains and two identical light chains, each of which has two different sections: the variable (V) and constant (C) regions. IgG’s Fab sections, particularly the VL and VH domains, are responsible for antigen recognition. These represent the top ends of the Y-shaped structure’s fork, whereas the Fc region is made up of the CH2 and CH3 domains, which represent the Y shape’s stem. The latter mediates its effector functions via interactions with immune cell Fcγ receptors (FcγRs) and the complement component C1q [37].

FccRs are classified as glycosylphosphatidylinositol (GPI)-anchored (FcγRIIIb), inhibitory (Fcγ RIIb), activating (FcγRI, FcγRIIa (FcγRIIc expressed by some individuals), and FcγRIIIa).

FcγRI (CD64) is expressed by myeloid cells (monocytes, dendritic cells, macrophages) and granulocytes (neutrophils, mast cells). Its stimulation leads to the activation and differentiation of monocytes towards monocyte-derived DCs and may contribute to antigen presentation to T cells.

Complement activation begins when C1q, ficolins or mannose-binding lectins bind to target surfaces like infected cells or microbes. IgG can participate in the activation of the complement system through three main mechanisms: 1—C1q strongly binds to IgG that has already attached to an antigen, but not to individual IgG molecules. 2—IgG molecules with an N-glycan on their Fc region terminating in mannose can also interact with mannose-binding lectin. 3—Finally, C3b, a later component of the complement cascade, can directly bind to IgG [38,39].

Among IgG subclasses, IgG4 exhibits a set of properties distinct from other IgG subclasses, which has contributed to its characterization as an anti-inflammatory, “benign” antibody potentially beneficial in allergic diseases. IgG4 plays also an important role in IgG4 autoimmune diseases (IgG4-AIDs), in tumour immunology and in IgG4-related diseases (IgG4-RDs) [40,41].

### IgG in Food Allergy and Oral Immunotherapy

It has been shown that this IgE-mediated cellular activation may be blocked by allergen-specific IgG, especially Ig4, through multiple mechanisms:

Blocking IgE activity through competition for allergen binding, thereby preventing degranulation of mast cells and basophils; Hence, because this epitope-specific interaction is a competitive one, it is largely promoted by high affinity and monovalency, in contrast to avidity which only plays a minor role in direct neutralization. Indeed, low-affinity IgG antibodies failed to neutralize allergens and did not block basophil/mast cell activation both in vitro as well as in vivo [42].

Engagement of the inhibitory receptor FcγRIIb which blocks IgE signal transduction. Although low-affinity IgG antibodies are unable to neutralize the allergen, they can still markedly reduce the activation of mast cells and basophils. This surprising effect relies entirely on FcγRIIb. For this mechanism to work, the low-affinity IgG must bind to allergen sites different from those recognized by IgE; only then can IgG interact with IgE already bound to FcεRI while also engaging the inhibitory FcγRIIb receptor. Lateral movement within the membrane and stabilization through avidity likely contribute, as weakly binding IgG needs to quickly associate with FcγRIIb to achieve effective inhibition [43].

Co-engagement of FcεRI and FcγRIIb by IgE-IgG-allergen immune complexes causes downregulation of receptor-bound IgE, resulting in desensitization of the cells. Both activation of FcεRI by allergen-specific IgE and engagement of FcγRIIb by allergen-specific IgG are driven by allergen-binding [19].

IgG blocks IgE-facilitated binding of allergen-IgE complexes to B cells, a key step needed for IgE-driven T-cell activation. In people with allergies, these allergen–IgE complexes attach to the low-affinity IgE receptor FcεRII (CD23) on B cells. Once activated, the B cells process the allergen and display its peptides on Human Leukocyte Antigen (HLA) class II molecules, along with the signals required to activate specific T-cell clones. This leads to T-cell activation, growth, and cytokine release. Studies have shown that serum from patients treated with birch or grass pollen immunotherapy can block this IgE-facilitated presentation of allergen by B cells to T-cell clones [44].

Further evidence for the inhibitory role of IgG4 comes from studies showing that removing peanut-specific IgG4 from the plasma of individuals undergoing OIT reduces the ability to suppress mast cell activation [45].

Chronic exposure to a specific allergen, either due to allergen-specific immunotherapy or regular exposure through food consumption, can induce the production of regulatory cytokines like IL-10, which drive class switching in B cells toward IgG, blocking IgE-mediated response. This interruption of the inflammatory feedback loop promotes the establishment of immune tolerance [46,47].

Evidence from the Learning Early About Peanut allergy (LEAP) trial found that early introduction of peanuts can help prevent peanut allergy by encouraging the development of peanut-specific IgG4 antibodies. In contrast, children who completely avoided peanuts were more likely to develop peanut-specific IgE [48].

Furthermore, growing evidence highlights IgG4 as an important marker of immunological shifts toward tolerance. Those findings suggest that in children with peanut or cow’s milk allergy, levels of allergen-specific IgG4 may help distinguish those whose allergy is likely to persist from those moving toward tolerance. In peanut allergy, higher sIgG4 over time are associated with natural resolution [49]. A similar pattern is seen in cow’s milk allergy: the IgG4/IgE ratio is higher in children who naturally outgrow the allergy, supporting the use of these antibody profiles as indicators of progression toward tolerance [50].

During OIT, while allergen-specific IgE initially rises, allergen-specific IgG—particularly IgG4—steadily increases over the course of therapy and becomes the predominant isotype. This shift leads to a higher sIgG/sIgE ratio, which has been associated with reduced clinical reactivity and improved tolerance to the allergen. Studies in peanut, cow’s milk, and egg OIT have consistently shown that patients who achieve sustained unresponsiveness typically exhibit a marked increase in the sIgG/sIgE ratio compared with those who remain reactive. Therefore, the development of higher sIgG/IgE ratios may be more likely to predict lasting tolerance than the absolute quantity of IgG4 [51,52].

Interestingly, despite allergen-specific IgG levels, particularly IgG4, rising in all participants during treatment, it has been shown that their levels remain elevated only in those who achieve a long-lasting response [53].

Detailed investigation of these IgG4-class antibodies demonstrated that, following OIT, the clinically relevant fraction of allergen-specific IgG4 appears to consist of neutralizing antibodies (nAb) that recognize specific conformational epitopes. IgG4 nAb levels increased significantly only in patients who maintained long-term responsiveness, further highlighting their role in the maintenance of SU [54].

However, unlike IgE, baseline serum IgG4 levels are not reliable predictors of clinical outcomes following OIT [52].

Additionally, Investigation of antigen-specific immunoglobulin responses at the oral mucosa during peanut oral immunotherapy revealed that peanut OIT led to significant increases in peanut-specific IgG4 (s-IgG4) in saliva, but after stopping therapy, peanut-sIgG4 levels dropped sharply. The ratio of peanuts-IgG4 to total IgG4 rose sharply during the build-up phase, remained stable during maintenance, and declined during the avoidance phase [55].

Although IgG4 plays a protective role in IgE-mediated food allergy, some studies have reported that IgG4 may contribute directly to tissue inflammation and the development of eosinophilic esophagitis, a potential adverse effect observed in the context of OIT. it has been shown that in active eosinophilic esophagitis (EoE), IgG4 forms immune complexes with food antigens, which accumulate within the esophageal tissue. In EoE, IgG4-positive plasma cells have been detected near active disease sites; these observations suggest a potential mechanism by which IgG4 may contribute to disease progression, potentially by forming immune complexes at high local concentrations. However, the evidence remains inconsistent, and further research is needed to clarify this association [56,57].

Recent developments in the field of OIT have studied the importance of other IgG subclasses and the classification of IgG1 as an equally dominant IgE-blocking antibody as IgG4. Allergen-specific IgG1 antibodies have been shown to inhibit allergen-induced basophil activation more effectively than IgG4. Previous studies also indicate that IgG1 is the predominant isotype during the early stages of the immune response in allergen immunotherapy. This suggests that the IgE-blocking activity of IgG1 may dominate initially, while the protective effect of IgG4 becomes more prominent during later phases of allergen immunotherapy (AIT). However, other studies have shown that removing IgG4 from sera collected after two years of AIT does not eliminate IgE inhibition, indicating a sustained protective role for IgG1 [58].

Moreover, the presence of high serum levels of allergen-specific IgG1 after the rush phase of OIT has been proposed as a potentially suitable biomarker for positive immune responsiveness to OIT [25].

Furthermore, analysis of B-cell Immunoglobulin Heavy chain (IGH) gene expression revealed notable patterns. In desensitized patients, only IGH for IgG2 expression was elevated. In contrast, individuals achieving SU showed significant upregulation of genes encoding the heavy chains of IgG4, IgG1 [50].

Other subclasses of the IgG family, including IgG1, IgG2, have also been investigated in the course of AIT. However, there have been few such studies, and each included only a few patients, and the precise responses of such sIgG subclasses during OIT still remain to be clarified [59]. The main findings are summarized in Table 2.

## 5. Immunoglobulin A (IgA)

IgA is the predominant antibody class at mucosal surfaces and is specialized for immune protection at epithelial barriers. It exists in two forms: monomeric IgA found in serum and polymeric secretory IgA (SIgA) present at mucosal sites.

In humans, two subtypes of IgA exist, IgA1 and IgA2. IgA1 differs from IgA2 by the presence of an additional 13 amino acids in the hinge region [60].

Secretory IgA (SIgA), synthesized by plasma cells within mucosal tissues such as the gut-associated lymphoid tissue (GALT), plays a crucial role in maintaining intestinal homeostasis by neutralizing pathogens through immune exclusion [61].

SIgA antibodies are produced and secreted at the highest levels at mucosal surfaces, including those of the gastrointestinal, urogenital, and respiratory tracts, while circulating IgA antibodies are present at comparatively lower concentrations in the bloodstream. IgA production in the gut is orchestrated by multiple immunological and metabolic signals within the gut-associated lymphoid tissue (GALT). TGF-β is a key driver of both T cell-dependent and independent class switching to IgA1 and IgA2, acting synergistically with cytokines such as IL-4, IL-5, IL-6, IL-10, and IL-21, as well as molecules like nitric oxide and retinoic acid. Retinoic acid, produced by CD103+ dendritic cells from dietary vitamin A, further supports IgA class switching, B cell differentiation, and migration of IgA+ plasma cells to the lamina propria. IL-21, mainly secreted by T-follicular helper cells, promotes IgA1-biased switching and can suppress IgE production, suggesting potential for allergy modulation. Additionally, B-cell-activating factor (BAFF) and A Proliferation-Inducing Ligand (APRIL), derived from intestinal epithelial cells, macrophages, and stromal cells, enhance B-cell activation, survival, and differentiation into IgA+ plasma cells, with APRIL also favoring IL-10-producing regulatory B cells. Together, these factors tightly regulate IgA responses in the gut and represent promising targets for therapeutic modulation of allergic immunity [60,61].

### IgA in Food Allergy and Oral Immunotherapy

Compared with IgG, the role of IgA in food allergy is less clearly defined and has been explored to a more limited extent. The role of IgA in the development of atopic disorders has been investigated by examining patients with selective IgA deficiency. Results have been mixed but overall show that those with IgA deficiency or a delay in IgA production (due to immune system immaturity) are at an increased risk of atopic disorders [62].

Secretory IgA (SIgA) is thought to limit allergic responses by reducing the uptake of food antigens in the gut, dampen immune activation by inhibiting chemotaxis of inflammatory cells and reducing IgE-mediated histamine release, suggesting that elevated mucosal SIgA may help prevent allergen–IgE interactions and subsequent symptoms. Additional evidence for SIgA’s protective potential comes from studies showing that allergen-specific SIgA can bind mast cells and basophils and inhibit IgE-dependent activation [15,46].

Maternal SIgA may also contribute to early immune protection from infections and potentially preventing food allergies such as cow’s milk allergy. Higher levels of total IgA in breast milk have been associated with a reduced risk of CMA and atopic dermatitis in early childhood, regardless of allergen specificity. Moreover, early-life intestinal and serum IgA also appear to correlate with lower susceptibility to IgE-mediated allergic diseases [63,64].

However, in some cases, plasma allergen-specific IgA was even higher in allergic children than in non-allergic controls. Moreover, stool peanut-specific IgA, used as a surrogate for small intestinal IgA, does not distinguish between allergic and nonallergic infants, nor does it predict current tolerance or future allergy.

Furthermore, the gut peanut-specific IgA epitope specificity is similar between nonallergic and peanut-allergic individuals, suggesting a common mechanism of induction [65].

These discrepancies suggest that IgA’s contribution varies depending on sampling site, timing, and patient atopic status, and that IgA alone is unlikely to serve as a reliable marker of tolerance.

As has been shown in the case of IgG, the induction of food unresponsiveness during OIT is frequently associated with rises in allergen-specific IgA, a pattern observed in OIT studies for peanut, cow’s milk, and egg. More specifically, the IgA2 isotype is increased in OIT treatment responders compared to non-responders. IgA2 is more predominant at mucosal sites than in serum and is more stable than IgA1 and may reflect a local IgA response in the gastrointestinal mucosa during OIT [51,66].

Since IgA class switching is driven by tolerogenic cytokines such as TGF-β and IL-10, increases in allergen-specific IgA could indicate a shift toward tolerogenic immune responses. Following egg-OIT, baseline comparisons showed that desensitized individuals had significantly higher serum egg white and ovalbumin-specific IgA levels than the failure group, consistent with reports of lower egg white-specific IgA2 in egg-allergic versus non-allergic individuals [25].

As observed for IgG, post OIT analysis of B cell IGH gene expression revealed that individuals in remission exhibited significant upregulation of genes encoding the heavy chains of IgA1and IgA2, supporting their protective role in food allergy [50].

Smeekens et al. observed significant rises in peanut-specific salivary IgA and sIgA/total IgA ratios in young children enrolled in the IMPACT trial of peanut oral immunotherapy (PnOIT). Children aged 12–48 months with confirmed peanut allergy received either PnOIT or placebo for 134 weeks and were evaluated for remission after 6 months of avoidance. In the PnOIT group, salivary peanut-specific IgA rose sharply early in treatment compared with placebo, showing a strong mucosal response to daily peanut exposure. This early rise reached a plateau, suggesting a threshold for how much antigen-specific IgA can be produced at the oral mucosa. Peanut-specific IgA was rarely detectable in serum, indicating that the immune response was primarily local. These findings support the idea that salivary IgA may contribute to desensitization and remission by limiting peanut absorption at the gastrointestinal surface, consistent with its role in immune exclusion. When examining clinical outcomes, the remission group did not show higher salivary IgA than other groups. This suggests that the increase in IgA during treatment, rather than the absolute level, is what aligns with successful desensitization. Surprisingly, the group that ultimately did not achieve desensitization showed higher salivary IgA at baseline. One interpretation is that very high mucosal IgA could bind and sequester peanut antigen, preventing its uptake by tolerogenic immune cells needed for inducing tolerance. Another possibility is that elevated baseline salivary IgA reflects underlying mucosal inflammation, which may hinder effective oral immunotherapy. Overall, high initial mucosal IgA may signal a lower likelihood of achieving tolerance [55]. Although salivary IgA was assumed to reflect gastrointestinal mucosal IgA, experimental confirmation was lacking. Furthermore, this hypothesis was not supported by studies on peanut allergy, showing that gut peanut-specific IgA subtype does not correlate with protection from allergy [65].

Overall, several studies described a modest or transient increase in serum allergen-specific IgA during OIT, whereas others did not observe significant changes over time. At the mucosal level, more consistent findings were observed. Multiple studies reported an increase in allergen-specific secretory IgA in saliva during OIT, particularly during intermediate or later phases of treatment. Similarly, a limited number of studies assessing intestinal immune responses described increased levels of allergen-specific IgA in fecal samples during OIT, suggesting enhanced mucosal immune exclusion at the gastrointestinal level. Changes in mucosal IgA were not always paralleled by corresponding alterations in serum IgA, supporting the concept of a compartmentalized immune response. However, the magnitude, timing, and clinical relevance of plasma, salivary and fecal IgA induction varied across studies, and their association with long-term clinical outcomes, including sustained unresponsiveness, remains incompletely defined. Those results are summarized in Table 3.

## 6. Discussion

Oral immunotherapy (OIT) represents a disease-modifying approach in the management of IgE-mediated food allergy, capable of reprogramming humoral immune responses and modulating both allergen-specific IgE levels and their downstream effector pathways [25]. During the early stages of OIT, allergen-specific IgE (sIgE) levels, particularly those directed against major allergen components, often rise; however, this transient increase does not correlate with heightened clinical reactivity, reflecting a dissociation between immunologic sensitization and symptomatic expression. Over the course of continued therapy, sIgE levels gradually decline, while total IgE generally remains stable, indicating that sIgE kinetics, rather than absolute IgE concentrations, are more informative for assessing therapeutic response. Importantly, the ratio of sIgE to total IgE and the qualitative characteristics of IgE—such as epitope specificity and antibody affinity—have emerged as stronger predictors of clinical outcomes than sIgE levels alone [5,50]. Patients who achieve sustained unresponsiveness often present with lower baseline sIgE, restricted epitope recognition, and lower sIgE/total IgE ratios, highlighting the value of detailed immunoprofiling in patient stratification prior to OIT initiation. Complementing these findings, studies have demonstrated that non-invasive mucosal measurements, such as salivary sIgE, can further inform individualized risk assessment, reflecting local immune responses and correlating with reaction thresholds during oral food challenges [10,48].

Parallel to changes in IgE, allergen-specific IgG, particularly IgG4, steadily increases during OIT and becomes the predominant protective isotype over time. This shift results in higher sIgG/sIgE ratios, which have been consistently associated with reduced clinical reactivity and improved tolerance across studies in peanut, cow’s milk, and egg allergy [16,49]. Neutralizing IgG4 antibodies, particularly those targeting conformational epitopes relevant for IgE binding, appear to play a crucial role in maintaining long-term SU. Early IgG1 responses also contribute to IgE-blocking activity, with IgG4 gradually dominating during the maintenance phase, suggesting a dynamic interplay between IgG subclasses in the modulation of allergic responses [54,58]. While IgG4 is largely protective, there is emerging evidence that in certain contexts, such as eosinophilic esophagitis, high local concentrations of IgG4 can form immune complexes with food antigens, potentially contributing to tissue inflammation, highlighting the need for careful monitoring and further investigation into subclass-specific effects [56].

Mucosal and systemic allergen-specific IgA, particularly the IgA2 subclass, also plays a supportive role in the induction of tolerance during OIT. Secretory IgA may limit allergen absorption at the gastrointestinal surface, inhibit IgE-mediated effector cell activation, and promote immune exclusion, thereby fostering a more tolerogenic environment. Increases in allergen-specific IgA are frequently observed in OIT responders, particularly at mucosal sites such as saliva, and appear to reflect local immune adaptation to repeated antigen exposure. Nevertheless, baseline IgA levels and post-treatment changes show considerable variability, influenced by factors including age, atopic status, and sampling site, and the correlation with clinical outcomes remains inconsistent. While IgA alone may not serve as a reliable biomarker for predicting SU, it likely contributes synergistically with IgG and IgE modulation to the overall immunologic reprogramming induced by OIT [15,25,55].

Taken together, these observations illustrate that OIT orchestrates a coordinated remodeling of humoral immunity, as summarized in Table 4. The early rise and subsequent decline of sIgE, the progressive accumulation of allergen-specific IgG—particularly neutralizing IgG4 antibodies—and the enhancement of mucosal IgA responses collectively underpin the induction of desensitization and, in many cases, the establishment of sustained unresponsiveness. Adjunctive strategies such as the use of omalizumab, which neutralizes circulating IgE and downregulates high-affinity IgE receptors, can further accelerate desensitization, improve safety during dose escalation, and potentially enhance long-term tolerance [35]. Overall, a comprehensive understanding of these dynamic immunologic changes, including the balance of antibody subclasses, epitope specificity, and mucosal responses, is critical for optimizing OIT protocols, improving patient stratification, and ultimately achieving durable clinical outcomes in food allergy.

## 7. Future Directions

Future research must concentrate on clarifying how baseline immunoglobulin profiles and their early evolution during oral immunotherapy (OIT) can be leveraged to stratify patient risk and personalize treatment pathways. Increasing evidence suggests that initial allergen-specific IgE levels, the ratio of IgG4 to IgE, and the presence of mucosal IgA may reflect meaningful biological heterogeneity among children undergoing OIT. However, their clinical application remains only partially explored.

A deeper characterization of these humoral trajectories could enable the identification of distinct responder phenotypes. These profiles may ultimately help determine which patients are suitable for conventional high-dose OIT, which may benefit from slower, low-dose protocols, and which may require adjunct biologic therapy to reduce risk and enhance tolerability. Ultimately, integrating immunoglobulin-based risk stratification with protocol selection and biologic add-on therapy represents a promising route toward a more individualized, mechanism-driven approach to food allergy treatment. Robust longitudinal studies—with harmonized measures of IgE, IgG, and IgA—will be essential to determine the predictive value of these biomarkers and to translate them into practical clinical algorithms capable of optimizing OIT outcomes and safety.

## Figures and Tables

**Table 1:** Allergen-specific IgE dynamics during OIT. Overview of serum and mucosal IgE parameters evaluated in OIT studies.

Parameter	Compartment	Observed Change During OIT	Notes on Clinical Relevance
Allergen-specific IgE (sIgE)	Serum	Transient increase during early phases, followed by gradual decline	Early increase does not correlate with increased clinical reactivity
Total IgE	Serum	Generally stable	Less informative than sIgE kinetics
sIgE/total IgE ratio	Serum	Progressive reduction over time	Lower ratios associated with desensitization and sustained unresponsiveness
Epitope specificity	Serum	Restriction of epitope recognition	Associated with favorable clinical outcomes
IgE affinity	Serum	Decrease in high-affinity IgE	Reflects qualitative remodeling of IgE responses
Salivary sIgE	Saliva	Variable changes	Non-invasive marker reflecting local immune activation and reaction thresholds

**Table 2:** Allergen-specific IgG dynamics during OIT. Overview of serum IgG parameters evaluated in OIT studies.

Parameter	Compartment	Observed Change During OIT	Notes on Clinical Relevance
Allergen-specific IgG (total)	Serum	Progressive increase	Associated with reduced clinical reactivity
IgG1 subclass	Serum	Early increase	Contributes to early IgE-blocking activity
IgG4 subclass	Serum	Robust and sustained increase	Predominant protective isotype; strongly associated with desensitization and SU
sIgG/sIgE ratio	Serum	Marked increase over time	Consistent biomarker of improved tolerance
Epitope overlap with IgE	Serum	Increased targeting of IgE-relevant epitopes	Supports blocking antibody function

**Table 3:** Allergen-specific IgA dynamics during OIT. Overview of serum, saliva and fecal IgG parameters evaluated in OIT studies.

Parameter	Compartment	Observed Change During OIT	Notes on Clinical Relevance
Allergen-specific IgA	Serum	Modest or variable changes	Inconsistent correlation with clinical outcomes
Secretory IgA (sIgA)	Saliva	Frequently increased in responders	Mucosal immune adaptation
Allergen-specific IgA	Feces (gut)	Increased in a subset of studies	Suggests enhanced immune exclusion at the intestinal level
IgA2 subclass	Mucosal sites	Preferential involvement	Associated with gastrointestinal immune defense

**Table 4:** Overview of immunoglobulin classes modulated during oral immunotherapy (OIT).

Immunoglobulin	General Trend During OIT	Functional Relevance
IgE	Early transient increase followed by gradual decline	Mediates allergic sensitization and immediate hypersensitivity reactions
IgG	Progressive increase, particularly IgG4	Acts as blocking antibody, reduces clinical reactivity, supports desensitization
IgA	Variable, often increased at mucosal sites	Supports mucosal tolerance, immune exclusion, and local protection

## Data Availability

No new data were created or analyzed in this study.

## Abbreviations

The following abbreviations are used in this manuscript:

Immunoglobulin E/G/A/D/M	IgE, IgG, IgA, IgD, IgM
Sustained Unresponsiveness	SU
Oral immunotherapy	OIT
High-affinity IgE receptor I	FcεRI
Specific-IgE/IgG/IgA	sIgE/sIgG/IgA
Interleukin 4/5/6/10	IL-4, IL-5, IL-6, IL-10
Transforming growth factor beta	TGF-β
Low affinity receptor for Ig	FcγRIIB
Group 2 innate lymphoid cells	ILC2s
Dendritic cell	DC
T helper 2	Th2
Cow’s milk	CM
Human Leukocyte Antigen	HLA
Neutralizing antibodies	nAb
Eosinophilic esophagitis	EoE
Immunoglobulin Heavy chain	IGH
Gut-associated lymphoid tissue	GALT
Allergen immunotherapy	AIT
B cell activating factor	BAFF
Proliferation-Inducing Ligand	APRIL
Secretory IgA	SIgA
Peanut oral immunotherapy	PnOIT
